# Longitudinal study across SARS-CoV-2 variants identifies transcriptionally active microbes (TAMs) associated with Delta severity

**DOI:** 10.1016/j.isci.2023.107779

**Published:** 2023-08-29

**Authors:** Priti Devi, Pallawi Kumari, Aanchal Yadav, Bansidhar Tarai, Sandeep Budhiraja, Uzma Shamim, Rajesh Pandey

**Affiliations:** 1 Division of Immunology and Infectious Disease Biology, INtegrative GENomics of HOst-PathogEn (INGEN-HOPE) Laboratory, CSIR-Institute of Genomics and Integrative Biology (CSIR-IGIB), Mall Road, Delhi 110007, India; 2 Academy of Scientific and Innovative Research (AcSIR), Ghaziabad 201002, India; 3 Max Super Speciality Hospital (A Unit of Devki Devi Foundation), Max Healthcare, Delhi 110017, India

**Keywords:** Microbiology, Virology

## Abstract

Emergence of new SARS-CoV-2 VOCs jeopardize global vaccine and herd immunity safeguards. VOCs interactions with host microbiota might affect clinical course and outcome. This longitudinal investigation involving Pre-VOC and VOCs (Delta & Omicron) holo-transcriptome based nasopharyngeal microbiome at taxonomic levels followed by metabolic pathway analysis and integrative host-microbiome interaction. VOCs showed enrichment of *Proteobacteria* with dominance of *Pseudomonas*. Interestingly*, Proteobacteria* with superiority of *Pseudomonas* and *Acinetobacter*, were highlights of Delta VOC rather than Omicron. Common species comprising the core microbiome across all variants, reiterated the significance of *Klebsiella pneumoniae* in Delta, and its association with metabolic pathways enhancing inflammation in patients. Microbe-host gene correlation network revealed *Acinetobacter baumannii*, *Pseudomonas stutzeri*, and *Pseudomonas aeuroginosa* modulating immune pathways, which might augment clinical severity in Delta. Importantly, opportunistic species of *Acinetobacter*, *Enterococcus*, *Prevotella*, and *Streptococcus* were abundant in Delta-mortality. The study establishes a functional association between elevated nasal pathobionts and dysregulated host response, particularly for Delta.

## Highlights

• Compositional differences in TAMs associated with Pre-VOC, Delta, and Omicron variants
• Deviation in Delta with opportunistic species of *Pseudomonas* and *Acinetobacter*
• Opportunistic species of *Acinetobacter, Enterococcus, and Prevotella* mark Delta mortality
• Host-microbiota networks define observed severity in Pre-VOC, Delta, and Omicron

## Graphical abstract

**Figure:**
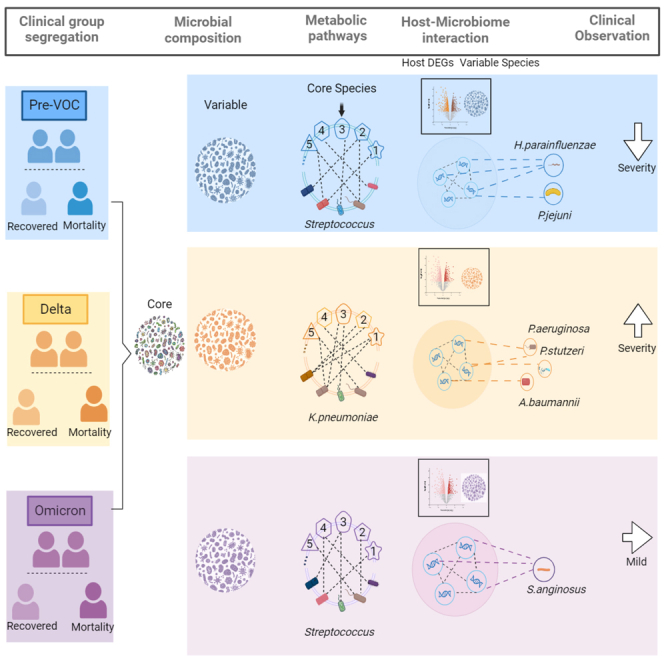

## Introduction

The SARS-Coronavirus-2 (SARS-CoV-2) outbreak in late 2019 caused a huge ordeal by adversely affecting the public health and economy worldwide, and was declared a COVID-19 pandemic in March 2020 by the World Health Organization (WHO).^1^ SARS-CoV-2, like other RNA viruses, has a proclivity for mutations during replication. The pandemic, thus, resulted in the rapid emergence and spread of several SARS-CoV-2 variants, wherein substantial studies have been carried out to understand the viral pathophysiology, evolution, and host response. In this regard, the routine monitoring of emerging SARS-CoV-2 variants through genomic surveillance assisted us to determine that thousands of mutations have been accumulating over time, globally and locally, in the viral genome.^2^ Because of their potential to induce increased transmissibility or virulence, some of these variants were designated as variants of interest (VOI) or variants of concern (VOC). Among the numerous variants discovered, the four major VOCs, Alpha (B.1.1.7), Beta (B.1.351), Delta (B.1.617.2), and Omicron (B.1.1.529), dominated the pandemic period. These emerging SARS-CoV-2 variants pose a threat, as an evolution of several variants may escape the immune system and cause pandemic transmission.^3^

As a result, studies have demonstrated heterogeneity in disease severity at both the individual or population level of SARS-CoV-2 infected patients with the same variant or distinct variants. For example, Delta and Omicron were infectious compared to wild or other variants of the virus, but Omicron infected patients have been reported to be less severe compared to Alpha and Delta in terms of viral loads, hospitalization, respiratory supplementation, and mortality.^4^ Omicron elicited high transmission rates and mild illness severity, even though both Omicron and Delta have been shown to escape *in vivo* neutralization.^5^^,^^6^^,^^7^ Besides the viral genotype, host factors such as age, comorbidity, gender, immune system, and demographics, have also been reported as a driving force in defining the disease phenotypes.^8^^,^^9^^,^^10^

Yet another candidate disease modulator, the respiratory tract microbiome, has recently been highlighted to influence the COVID-19 disease severity and outcome. Since the nasal and oral cavities are the SARS-CoV-2’s entrance routes, the microbial populations, especially those of the respiratory and gastrointestinal tracts, are likely to altered. Several genera, including *Veillonella*, *Acinetobacter*, *Klebsiella*, *Prevotella*, *Gemella*, *Streptococcus*, *Haemophilus*, and *Moraxella*, are abundant and linked to SARS-CoV-2 disease severity in the upper respiratory tract.^11^^,^^12^ Moreover, our lab’s recent study identified a significant differential abundance of transcriptionally active microbes (TAMs) across different COVID-19 severity groups, including *Achromobacter xylosoxidans* and *Bacillus cereus* in the mortality, *Leptotrichia buccalis* in the severe, *Veillonella parvula* in the moderate, and *Actinomyces meyeri* and *Halomonas* sp. in the mild patients. In yet another study by our group, the importance of commensal species including *Streptococcus salivarius*, *Streptococcus dysgalactiae*, and *Pseudomonas alcaliphila* were found in the milder presentation of the SARS-CoV-2 infected patients who received two doses of COVID-19 vaccine compared to unvaccinated & infected patients.^13^ Many studies have demonstrated the superiority of commensal microbial composition over opportunistic pathogens in the early recovery of COVID-19 patients.^14^^,^^15^^,^^16^ These findings suggest the importance of understanding the dynamics of TAMs in longitudinal studies with respect to the COVID-19 patients who were exposed to the virus during several waves, and if so, how this could be coupled to the severity of the disease and the patients' clinical outcomes.

Through an integrative approach of dual RNA-Sequencing (RNA-Seq) and metagenomic analysis, we explored and elucidated the role of TAMs across Pre-VOC, Delta, and Omicron in modulating clinical outcomes, i.e., recovery and mortality, for each group. Our analysis integrated and comprehensively evaluated the core microbiota along with variable species in terms of diversity, composition, and metabolic pathways associated with pertinent microbial species. We also integrated host response genes and variable microbial species from each group to gain new insights into host-microbiome dynamics and explored the associations between host pathways, genes, and microbial species. To help define the severity during the Delta period, this study accentuates the differential presence of pathobionts species in the Delta group which was later discovered to be more widely dispersed in the Delta mortality patients.

## Results

### Study design, sample segregation and clinical characteristics of the cohort, Pre-VOC, Delta, and Omicron

In the study, we evaluated the role of transcriptionally active nasopharyngeal microbiota during different waves of COVID-19 pandemic in India. Over a period of 2 years, between April 2020 and March 2022, thousands of samples of hospital admitted patients were collected for SARS-CoV-2 genome sequencing, using ONT (Oxford Nanopore Technology) platform. Sequence analysis led to the identification and timely reporting of different variants of SARS-CoV-2 circulating in the population. For this study, a subset of 214 patient samples based on their clinical data and SARS-CoV-2 variant/s, were selected from different time points coinciding with different waves of COVID-19. Samples collected between April-July 2020 were designated as Pre-VOC group (n = 125) while the Delta and Omicron wave samples are the VOC group (n = 89). Within the VOC, the Delta wave samples were collected between March and April 2021 whilst the Omicron between January and March 2022.

**Figure 1:**
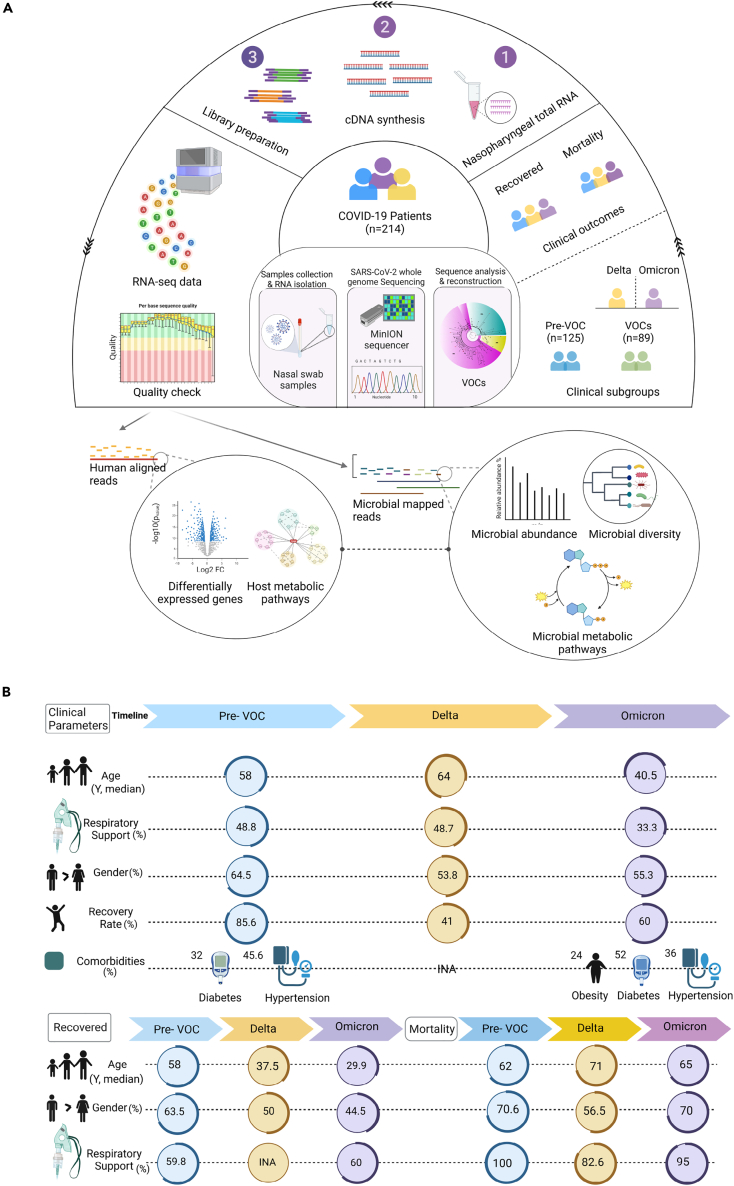
Study design and clinical data overview (A) Schematic figure depicts the study design and workflow of the experimental process for the COVID-19 patients of Pre-VOC and VOCs (Delta and Omicron), as well as the clinical outcome (Recovered and Mortality). RNA from nasal swabs used for viral genome sequencing on ONT, and dual RNA-Seq on Illumina NextSeq 2000 platform. Transcriptomic and metagenomic data analysis follows the functional integrative exploration between TAMs, DEGs and metabolic pathways. (B) Graphical representation of clinical parameters highlighting the median age, percentage of patients requiring respiratory support, gender distribution, recovery rates and comorbidities for the three COVID-19 waves, Pre-VOC, Delta, and Omicron. Additionally, the demographics for recovered and mortality subgroups have been illustrated.

The role of TAMs in different VOCs was also explored taking into consideration the clinical outcome i.e., recovered and deceased due to COVID-19. Holo-transcriptome, capturing both the human and the microbial RNA was sequenced using the library prepared from nasopharyngeal total RNA. The RNA sequenced data underwent rigorous quality check, after which the human-mapped and microbial-mapped reads were separated for performing different downstream analyses. The filtered human reads averaged 969230 across all patient samples whilst the average microbial reads obtained across patient samples was ≈5661395 (Table S1). Figure 1A demonstrates the experimental methodology including patient segregation, dual RNA-seq workflow, sample segregation, and downstream analysis. Microbial data were analyzed for taxonomic classification of the microbiota captured from the nasopharynx and species based metabolic profiling whereas host transcriptome data were utilized to identify differentially expressed genes (DEGs). For stringent analysis and inferences, three samples were removed due to the relatively low microbial reads.

Figure 1B highlights the clinical characteristics of the patients across the different variants. Demographic analysis suggests that the median age of the patients in the Pre-VOC (58 years) and Delta (64 years) were significantly higher (p=<0.05) than the Omicron (40.5 years) whereas gender (M/F ratio) among the three subgroups was comparable (non-significant). Around 48% of patients in Pre-VOC and Delta required respiratory support, which decreased to 33% in Omicron infected patients. Recovery rate was highest in the Pre-VOC (85.6%) followed by Omicron (60%). Delta showed the highest fatality with recovery rate falling to 41%. Comorbidities like hypertension and diabetes were present in both Pre-VOC and Omicron, although the data from Delta patients was not available. Segregation of patient samples from different wave time periods into recovered and mortality, showed that the median age of the recovered patients was significantly higher in the Pre-VOC when compared to Delta and Omicron whereas it was comparable in the mortality patients. A gender difference was significant (p=<0.05) amongst all three subgroups, Pre-VOC (63.5%), Delta (50%), and Omicron (44.5%) in the recovered patients whereas, in mortality patients, difference was observed only in the Delta (56%) with Pre-VOC and Omicron showing similar M/F ratio (70%). More than 80% of mortality patients from all subgroups required respiratory support whereas only 60% of recovered patients required the same. These features suggest that clinical severity was observed during the Delta variant infections with higher age group patients succumbing to COVID-19. Further, our study proceeded to resolve/capture the different SARS-CoV-2 variants' effect on the nasopharyngeal microbiota and its association, if any, with clinical outcomes.

### Evaluation of the nasopharynx TAMs composition across Pre-VOC, Delta and Omicron revealed highly variable niches amongst Pre-VOCs

**Figure 2:**
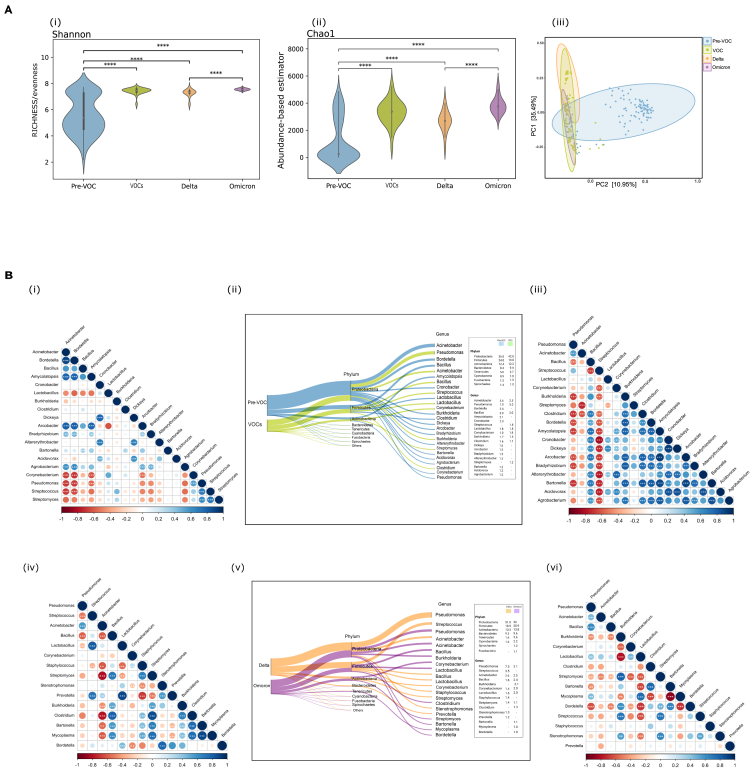
Diversity and abundance patterns in Pre-VOC and VOCs (A) Visualization of alpha and beta diversity. Violin plots representing (i) Shannon (Richness), (ii) Chao-1 (Abundance-based estimator) with p *values* calculated by the Kruskal Wallis test (‘∗∗∗∗’ indicates <0.0001), (iii) Beta diversity wherein Principal Coordinate Analysis (PCoA) shows the differential composition of the nasopharyngeal microbes across Pre-VOC, VOC, Delta, and Omicron. (B) Illustration of percent relative abundance proportion of phyla and genera identified in (ii) Pre-VOC, VOC and (v) Delta, Omicron. Spearman’s correlation plot highlight correlation between the relative abundances of bacterial genera across (i) Pre-VOC, (iii) VOC, (iv) Delta, and (vi) Omicron. Blue represents positive and red negative correlation, whereas larger size and darker color of circles depict strong correlation. Genera showing significant and strong correlation have been highlighted with ‘∗’, ‘∗∗’, ‘∗∗∗’ based on *pvalue* (<0.05, <0.01, <0.001) respectively calculated by Spearman’s Correlation.

We first investigated in our study subgroups whether different variants of SARS-CoV-2 affecting the population at different time periods were associated with changes in the taxonomy of the transcriptionally active microbial communities. Interestingly, there was a shift in the alpha diversity reflecting lower diversity (less enrichment in taxonomic groups) captured in Pre-VOC when compared to the VOCs (Delta and Omicron). Shannon richness and abundance-based Chao-1 indices were significant (p = 0.01) when Pre-VOC was compared to VOC (Delta and Omicron). Similarly, both alpha diversity indices were also significant when microbial diversity was compared between Delta and Omicron (p = 0.01). Thus, an increasing trend in the richness and abundance of transcriptionally active microbes (TAMs) was observed as we moved from Pre-VOC toward VOCs (Figures 2A-i and 2A-ii), and Table S2). Beta diversity analysis also showed distinct clustering patterns between the Pre-VOC and VOCs when visualized in a PCoA plot. Delta and Omicron although clustered together toward one side of the PCoA plot yet PC1 variance (35.5%) was higher with Delta and Omicron falling in different axes (Figure 2A-iii).

With significant differences in alpha and beta diversity between the SARS-CoV-2 variants, we analyzed the microbial composition variability at the phylum and genus level. The most abundant phylum, *Proteobacteria* showed a higher presence in VOCs (43% vs. 36%) whereas *Firmicutes* was higher in Pre-VOC (24% vs. 20%). *Actinobacteria* and *Bacteroidetes* showed near equal abundance in both Pre-VOC (12%; 8%) and VOC (12%; 9%) respectively. The nasopharyngeal microbiome is principally composed of the four above-mentioned phyla, yet, strikingly, *Tenericutes* normally found in less abundance, showed substantial presence in Pre-VOC (5%) compared to VOCs (3%). At the phylum level, the differences were spontaneous, but the genera level difference were very prominent between Pre-VOC and VOCs. Interestingly, we found >1% representation of several genera in Pre-VOCs, signifying a higher degree of variability in microbial diversity between Pre-VOC and VOCs. Pre-VOC was dominated by *Acinetobacter* (5%), *Bordetella* (3.4%), and *Bacillus* (3%), whereas VOCs comprised *Pseudomonas* (5%) and *Acinetobacter* (2.5%). Several genera (with greater than 1% abundance) were exclusively present in Pre-VOC as shown in Figure 2B(ii) and Table S3).

Spearman’s correlation analysis and co-occurrence networks (Figure S1) were created to study specific significant interactions between the different genera, which could help us understand the absence of a few prominent genera of nasopharyngeal microbiota across different subgroups. The correlation network emphasized a strong negative correlation (p = 0.01) of *Acinetobacter* and *Bordetella* (prominent genera) of Pre-VOC with *Corynebacterium*, *Pseudomonas*, *Streptococcus* and *Streptomyces*, possibly a reason for their less abundance in Pre-VOC (>1%). The exclusive genera of Pre-VOC like *Amycolatopsis*, *Acrobacter*, and *Agrobacterium* were positively correlated (p*=0.01*) with *Acinetobacter* and *Bordetella*, again signifying their abundance in the Pre-VOC group (Figure 2B-i). In VOCs, *Pseudomonas* and *Acinetobacter* were positively correlated with each other as well as with *Streptococcus*, justifying their >1% abundance in VOCs whereas, all three were in negative correlation with nearly all other genera (Figure 2B-iii).

Delving deeper to identify alterations in TAMs within VOCs, we observed that a higher trend of *Proteobacteria* in VOCs arose from its abundance in Delta (51%) as compared to Omicron (36%), whilst *Firmicutes* and *Bacteroidetes* were higher in Omicron (Figure 2B-v). Notably, *Pseudomonas* highly dominated Delta, whereas the Omicron did not show substantial dominance of any one genus (Figure 2B-v). Looking for co-occurrence (Figure S1) and correlation analysis, *Pseudomonas* in Delta showed a negative correlation with all the other genera except *Acinetobacter*, whereas *Streptococcus, Prevotella*, and *Lactobacillus* were in strong positive correlation with each other (Figure 2B-iv). Diverse single genus correlations were observed for Omicron, which did not indicate clear dominance of one or a few microbial species Figure 2B-vi. The interactions are summarized in Table 1.

**Table 1:** Summarizing the genera association across groups

Pre-VOC	VOCs	Delta	Omicron
Acinetobacter	Pseudomonas	Pseudomonas	Pseudomonas
Bordetella	Acinetobacter	Acinetobacter	Acinetobacter
Bacillus		Streptococcus	
Amycolatopsis	Streptoccocus	Lactobacillus	Mutiple +ve and –ve correlations
Arcobacter	Burkholderia	Prevotella	
Agrobacterium	Clostridium	-ve correlation with all other genera mentioned	
Pseudomonas	Bordetella		
Streptococcus	Bartonella		
Streptomyces	Amycolatopsis		
Corynebacterium	Bacillus		

Thick lines denote positive and dashed lines negative correlations.

Major correlations between microbial genera identified in Pre-VOC and VOCs (Delta and Omicron).

### Comprehensive evaluation of core and variable components of the TAMs associated with Pre-VOC, Delta, and Omicron

#### Differential abundance of commensal and opportunistic species contributes to core microbiota of Pre-VOC, Delta, and Omicron

**Figure 3:**
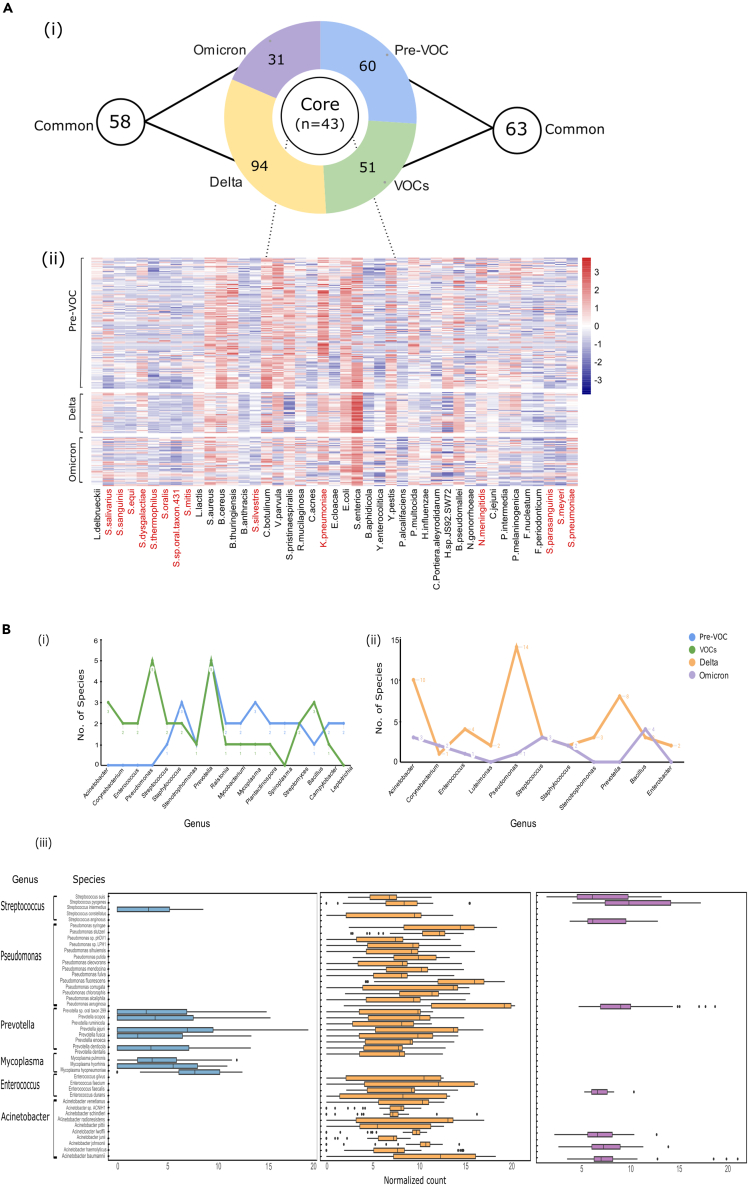
Illustration of core and variable microbial species profile across Pre-VOC, Delta and Omicron (A) Core microbial species spectrum across Pre-VOC, Delta, and Omicron. (i) Illustration depicting the distribution of core and variable microbial species across the SARS-CoV-2 variants. (ii) Heatmap represents the core microbiota across all groups wherein the abundance of *Streptococcus* species and common respiratory pathogens have been highlighted in red. (B) Variable microbial species representation for the Pre-VOC, VOC, Delta, and Omicron. Six prominent genera between (i) Pre-VOC and VOCs, (ii) Delta and Omicron, highlighting the variation in the number of species between the subgroups, and (iii) Abundance of the species of the six main genera across Pre-VOC, Delta, and Omicron are highlighted.

Next, we wanted to comprehend the differentially abundant microbial species, which were commonly and distinctively present across the variants, shedding light on potential dysbiosis and bacterial co-infection occurrence in the subgroups. For uniformity across all groups, the bacterial species with a relative cumulative abundance of 0.1% and presence in at least 50% of the samples within each group, were taken into consideration. Consequently, 103, 94, 137, and 74 bacterial species were retrieved from Pre-VOC, VOCs, Delta, and Omicron respectively. Pre-VOC and VOCs shared 63 common species whereas Delta and Omicron had 58 species in common. Together, 43 bacterial species were commonly present, designated as core microbiome across the variant subgroups associated with SARS-CoV-2 infection (Figure 3A-i). The heatmap highlights the differential abundance of common bacterial species across Pre-VOC, Delta, and Omicron (Figure 3A-ii). We observed that 11/43 were *Streptococcus* species, which were equally abundant across all variants. These species were found to be in strong positive correlation with each other as well as with *Lactococcus lactis* (probiotic commensal) in all three groups (Figure S2). The predominance of common potential respiratory pathogens like, *Streptococcus pneumoniae*, *Haemophilus influenzae*, *Staphylococcus aureus*, *Klebsiella pneumoniae*, were observed across all three variants, possibly alluding to a common occurrence during the SARS-CoV-2 infection. Differential abundance of a few species was also observed across Pre-VOC, Delta, and Omicron. *B. cereus*, *B. thuringiensis*, *Halomonas*, *C. botulinum*, *V. parvula* and *S. pristinaespiralis*, a mix of commensal and opportunistic pathobionts were abundant in Pre-VOC whereas *E. coli*, *S. enterica* and *B. pseudomallei* (opportunistic pathobionts) dominated the Delta (Figure 3A-ii). *Bacillus* species, *Halomonas*, and *C. botulinum* were specifically positively correlated with each other as well as *Streptococcus* species, in concurrence with their abundance in Pre-VOC. Contrarily, these microbial species were found to be in negative correlation in Delta as well as Omicron, signifying their reduced abundance in both (Figure S2). Interestingly, Pre-VOC demonstrated all positive correlations between species, whereas strong negative correlations amongst the core microbes were found in the VOCs of Delta and Omicrons, suggesting a heightened dysbiosis/disequilibrium in the VOCs.

#### Variable TAMs of Delta enriched with opportunistic pathobionts of Pseudomonas and Acinetobacter species

To further understand how different variants of SARS-CoV-2 infection could have perturbed the equilibrium between commensal/opportunistic bacteria, we expanded our investigation of the nasopharyngeal microbiota from core/common species to distinctly abundant species that comprised the variable microbiota associated with each group. Pre-VOC, VOCs, Delta, and Omicron subgroups harbored 60, 51, 94, and 31 variable species belonging to 43, 34, 51, and 17 genera, respectively. A total of 71 genera was detected across all four groups, Table S4. We focused on those genera that either were prominent genera of nasopharyngeal TAMs or showed variation in the number of species between two subgroups, Pre-VOC/VOCs, and Delta/Omicron. Accordingly, 17 genera were identified in Pre-VOC/VOC (Figure 3B-i) and 11 between Delta/Omicron (Figure 3B-ii).

The Pre-VOC variable microbiome was devoid of species belonging to *Acinetobacter, Corynebacterium, Enterococcus*, and *Pseudomonas*. Contrarily, it contained species from the genera *Staphylococcus, Mycoplasma*, *Mycobacterium*, and *Spiroplasma,* which either were commensals or not known to cause severe human infections (Figure 3B-i). The VOCs showed an enhanced presence of *Acinetobacter, Corynebacterium, Enterococcus, Pseudomonas*, and *Prevotella* (Figure 3B-i), which subsequently was observed to be differentially distributed between Delta and Omicron (Figure 3B-ii). Except for *Corynebacterium* species, which was equally present in Delta and Omicron, the rest of the genera were abundant only in the Delta. Further, the species captured from the top six genera (*Streptococcus, Pseudomonas, Prevotella, Mycoplasma, Enterococcus*, and *Acinetobacter*) across Pre-VOC, Delta, and Omicron, were investigated for their association with enhanced dysbiosis.

Strikingly, an abundance of most of the species was found to be in Delta followed by Pre-VOC and Omicron (Figure 3B-iii). Looking closely at the functional role of these variably present bacterial species, we concluded that *Streptococcus intermedius, Streptococcus constellatus,* and *Streptococcus anginosus* constituting the Streptococcus milleri group was distinctly present in Pre-VOC, Delta, and Omicron. Alternatively, *S. pyogenes* and *S. suis*, which are reported as potential pathogens, were found in Delta and Omicron only. A sharp delineation was observed for *Pseudomonas* and *Acinetobacter*, especially with *Pseudomonas aeruginosa*, *P seudomonas putida, P seudomonas fulva, Pseudomonas stutzeri*, and *P. mendocina,* which are known human pathogens and prevalent only in Delta. Similarly, *Acinetobacter* species, such as *Acinetobacter baumannii, Acinetobacter haemolyticus, Acinetobacter johnsonii, Acinetobacter junii,* and several others with the potential to cause human infections prevailed in Delta, signifying a highly opportunist dysbiotic microbiome during infection by Delta variant which could be one of the modulators of the observed disease severity during the Delta wave of infection, especially in India.

### Metabolic pathways enrichment analysis and integrative analysis of host gene expression and TAMs reveal functionally important TAMs for different variants

We next investigated the functional pathways associated with the active microbiome, which have the potential to alter immune profile and hence host response to infection. We analyzed the metabolic pathways against the MetaCyc database using HUMAnN3 generated microbial data for characterizing the metabolic pathways and their associated transcriptionally active microbial species for each subgroup. These TAMs were further combined with the host gene expression for integrative analysis, elucidating the host’s response to observed changes in the microbiota for Pre-VOC, Delta, and Omicron. We took a two-pronged approach wherein we dealt with the microbial species of the core and variable microbiome separately to demarcate the effects/consequences of either, on SARS-CoV-2 infection by different variants.

#### Core microbial species across Pre-VOC, Delta and Omicron reveals functionally active K. pneumoniae in Delta affecting host response

**Figure 4:**
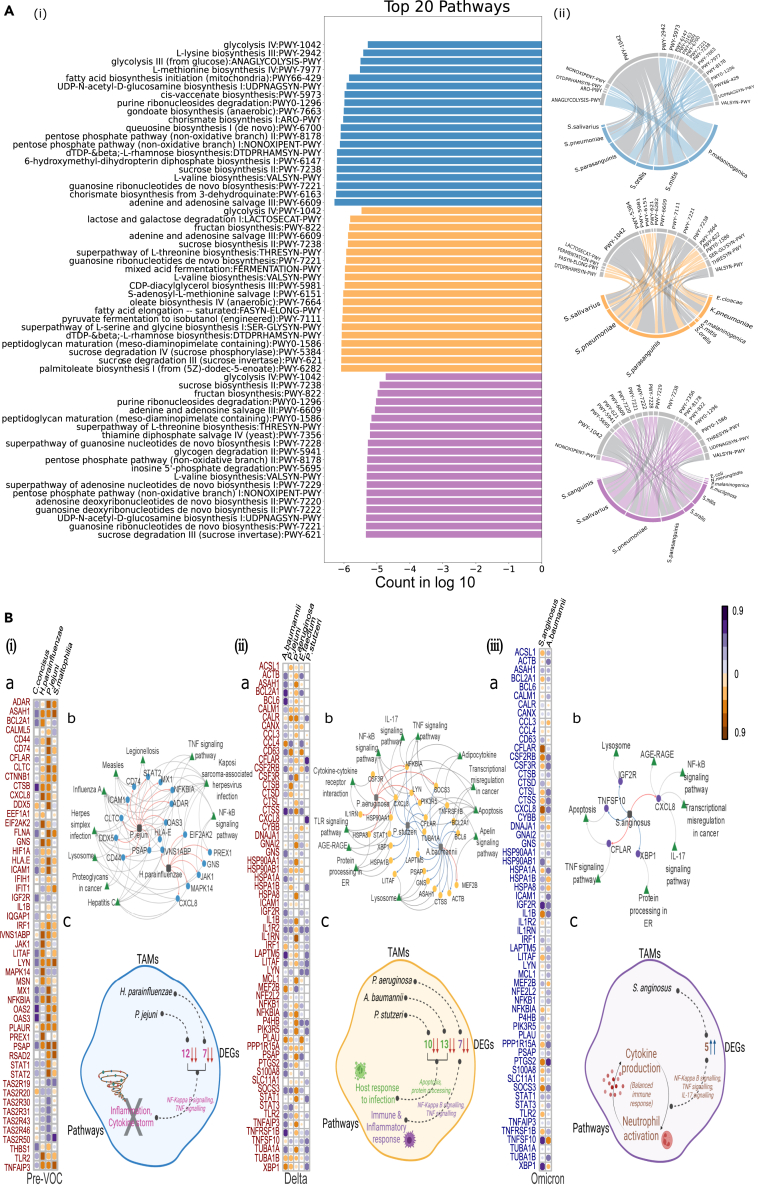
Association between microbial species, metabolic pathways and host genes (A) Metabolic pathway enrichment analysis of the core microbial species. (i) Top 20 enriched metabolic pathways in Pre-VOC, Delta and Omicron based on the MetaCyc database. (ii) Circos plot represents the enriched pathways associated with bacterial species for all the three groups, with the common pathways highlighted in gray color. (B) Microbiome and host transcriptome interaction analysis. Associations between the microbiome and transcriptome in, (i) Pre-VOC, (ii) Delta, and (iii) Omicron. (a) Correlation matrix of pairwise associations between the microbial species and their associated genes. Bacterial species are along the rows, and DEGs are along the columns. (b) Network of associations between TAMs (square nodes) with highly interacting genes (circular nodes) and their pathways (triangular nodes). Arrows indicate the positive (blue) or negative (red) correlation between the species and the genes. Gray arrows indicate relationships between DEGs and pathways. (c) Conceptual illustrations highlighting the biological inference from the association between TAMs, DEGs, and pathways.

The metabolic pathways that were selectively enriched for the 43 bacterial species (commonly present across all three subgroups, Figure 3B), were separately extracted for each of the three subgroups. We removed/filtered out those pathways from each of the subgroups which were represented by <20% of samples from that respective group. Finally, for the 43 common bacterial species, we obtained a set of 27, 145, and 117 metabolic pathways for Pre-VOC, Delta, and Omicron, respectively (Table S5). Figure 4A-i depicts the list of the top 20 enriched pathways in each of the respective groups, whilst Figure 4A-ii demonstrates the bacterial species associated with these metabolic pathways. Notably, 5 out of 20 pathways i.e., glycolysis IV (PWY-1042), adenine and adenosine salvage III (PWY-6609), sucrose biosynthesis II (PWY-7238), guanosine ribonucleotide *de novo* synthesis (PWY-7221) and L-valine biosynthesis (VALSYN-PWY) overlaid the three groups, Figure 4A-ii in gray shades.

These pathways characterize major metabolic functions of glycolysis, nucleotide synthesis, and amino acid synthesis, which are essentially required for bacterial growth, replication, quorum sensing, and quorum control. Sucrose biosynthesis II pathway plays a role to maintain osmotic balance and stabilize protein and membrane structure and function of the bacterial cell wall. Importantly, it is also required for biofilm formation. Notably, several *Streptococcus* species, which dominated the core microbiota, were seen to be metabolically active resulting in the enrichment of these pathways. Moreover, few pathways were uniquely present in each subgroup, 10 in Pre-VOC and Delta, and 7 in Omicron (Figure 4A and Table S6). Interestingly, only Delta harbored metabolic pathways, which were associated with the pathogenic respiratory bacterium *K. pneumoniae*. These pathways, (fatty acid elongation (FASYN-ELONG-PWY), mixed acid fermentation (FERMENTATION-PWY), oleate biosynthesis IV(PWY-7664), super-pathway of L-serine and glycine biosynthesis I (SER-GLYSYN-PWY), and palmitoleate biosynthesis I (PWY-6282) pathways) have been implicated in proinflammatory responses in the host. In Omicron, the dominance of Streptococcus species enriching the metabolic pathways was only observed.

#### Variable microbial species interaction with host genes corroborates with clinical severity observed for Pre-VOC, Delta, and Omicron

The next set of integrative analysis involved TAMs, which constituted the variable microbiome of Pre-VOC, Delta, and Omicron (Figure 4B). Since, the number of variable TAMs in each group were comparatively high (Pre-VOC = 60, Delta = 94, and Omicron = 31), we investigated those species that showed the highest metabolic activity. Like the previous analysis, we extracted for each group, all metabolic pathways associated with their variable TAMs, and removed those pathways from each of the groups which were represented by <20% of samples for that respective group (Table S5). This resulted in four, five, and two bacterial species in each subgroup. We then compared transcriptome profiles between VOC/pre-VOC and Omicron/Delta which identified 748 differentially expressed genes (DEGs) for VOC/pre-VOC and 891 DEGs in Omicron/Delta (FDR ≤0.05 and |log2 foldchange| ±2) (Table S7). To characterize the functional relevance of these DEGs, we analyzed for significantly enriched biological pathways (FDR ≤0.05) using Gene Set Enrichment analysis (GSEA) (Table S8). Significant pathways (p*= 0.01*) related to immune response and infectious disease and their associated DEGs (11 pathways & 53 genes for Pre-VOC/VOCs; 13 pathways & 60 genes for Delta/Omicron) were considered for microbiome-transcriptome interaction analysis in a two-step process. Firstly, we applied the Spearman measure to characterize strong correlations (correlation coefficient 0.8 & p *value ≤ 0.05*) between bacterial species and DEGs for Pre-VOC, Delta, and Omicron (Figure 4B-a).

DEGs, which showed a significant correlation with bacterial species in Pre-VOC and Delta, displayed downregulated expression, whereas the DEGs of Omicron were upregulated. The four bacterial species, *Campylobacter concisus, P. jejunii, Haemophilus parainfluenzae*, and *Stenotrophomonas maltophilia* of Pre-VOC showed distinct associations with different sets of genes*. P. jejunii* and *H. parainfluenzae* showed maximum interactions with 12 and 7 associated genes, respectively, that function primarily for antiviral response in infectious diseases. A few genes, as *NFKBIA, ICAM1, CXCL8*, and *TNFAIP3* are involved in NF-κB signaling and TNF signaling pathways, which have been known to cause inflammation and cytokine storms, augmenting COVID-19 severity. The negative association of *P. jejunii* and *H. parainfluenzae* might have been beneficial reflecting reduced severity in Pre-VOC when compared to the VOCs (Figure 4B-i). We looked across VOCs of Delta and Omicron, with the same set of genes, and associations with the differentiated bacterial species.

In Delta, maximum associations were captured for *A. baumannii* (13 genes), *P. stutzeri* (10 genes), and *P. aeruginosa* (7 genes). Highly pathogenic species, *P. aeruginosa* abundance was discovered to be negatively correlated with the genes *IL1RN* (inhibitor of interleukin-1), *NFKBIA* (inhibitor of NFKB), *SOCS3* (suppressor of cytokine signaling), *HSP90AA1* and *HSPA8* (regulates infection-induced inflammatory response), thus dysregulating major pathways such as TNF signaling, NF-κB, and IL-17 signaling. Thus, *P. aeruginosa* is found to contribute toward altering the immune and inflammatory response to SARS-CoV-2 infection, leading to higher observed severity in Delta. Contrarily, an abundance of *A. baumannii* and *P. stutzeri* species were discovered to be positively correlated with host genes. Apoptosis (*BCL6, CTSS, ACTB, TUBA1A, BCL2A1*), protein processing in endoplasmic reticulum (*HSPA1B, XBP1*), and lysosomes (*LAPTM5, PSAP, GNS, LITAF*) pathways regulated by these genes are responses to infection and injury (Figure 4B-ii).

Interestingly, the Omicron subgroup did not demonstrate a significant association between genes and bacterial species with *S. anginosus* showing five gene associations whereas *A. baumanni* with only one gene. Importantly, *S. anginosus*, a commensal, was positively correlated with *XBP1* (upregulate the transcription of inflammatory cytokines) and *TNFSF10* (TNF linked apoptosis inducer) whilst, negatively correlated with the *CXCL8* gene which is the primary cytokine involved in neutrophils recruitment at the site of infection. Thus, variable microbiota did not seem to majorly affect the immune response observed during Omicron infection, alluding to mild clinical presentation in the Omicron variant infected individuals (Figure 4B-iii). Next, we analogously constructed association networks using the aforementioned microbial species, their associated DE genes, and pathways, Figure 4B for Pre-VOC (4B-ib), Delta (4B-iib), and Omicron (4B-iiib). Figure 4B-c summarizes the gene-microbial association and its affected clinical pathways for Pre-VOC, Delta, and Omicron.

### Differential presence of TAMs in Pre-VOC recovered and Delta mortality highlights their putative role in clinical outcome

Considering our findings between Pre-VOC, Delta, and Omicron, we carefully examined TAMs segregating through clinical outcomes between the subgroups, recovered and mortality, to understand the plausible role or trend of microbial composition, if any, associated with recovery or mortality. We elucidated the comparative microbial dynamics of each variant’s recovery and mortality subgroups by analyzing alpha diversity (Shannon and Chao1 index) as well as phylum and genera level relative abundance (Figure S3, Tables S9, and S10). Importantly, we observed significant differences for alpha diversity between the variant’s recovery and mortality subgroups, with an exception for mortality subgroups of Pre-VOC and Delta.

**Figure 5:**
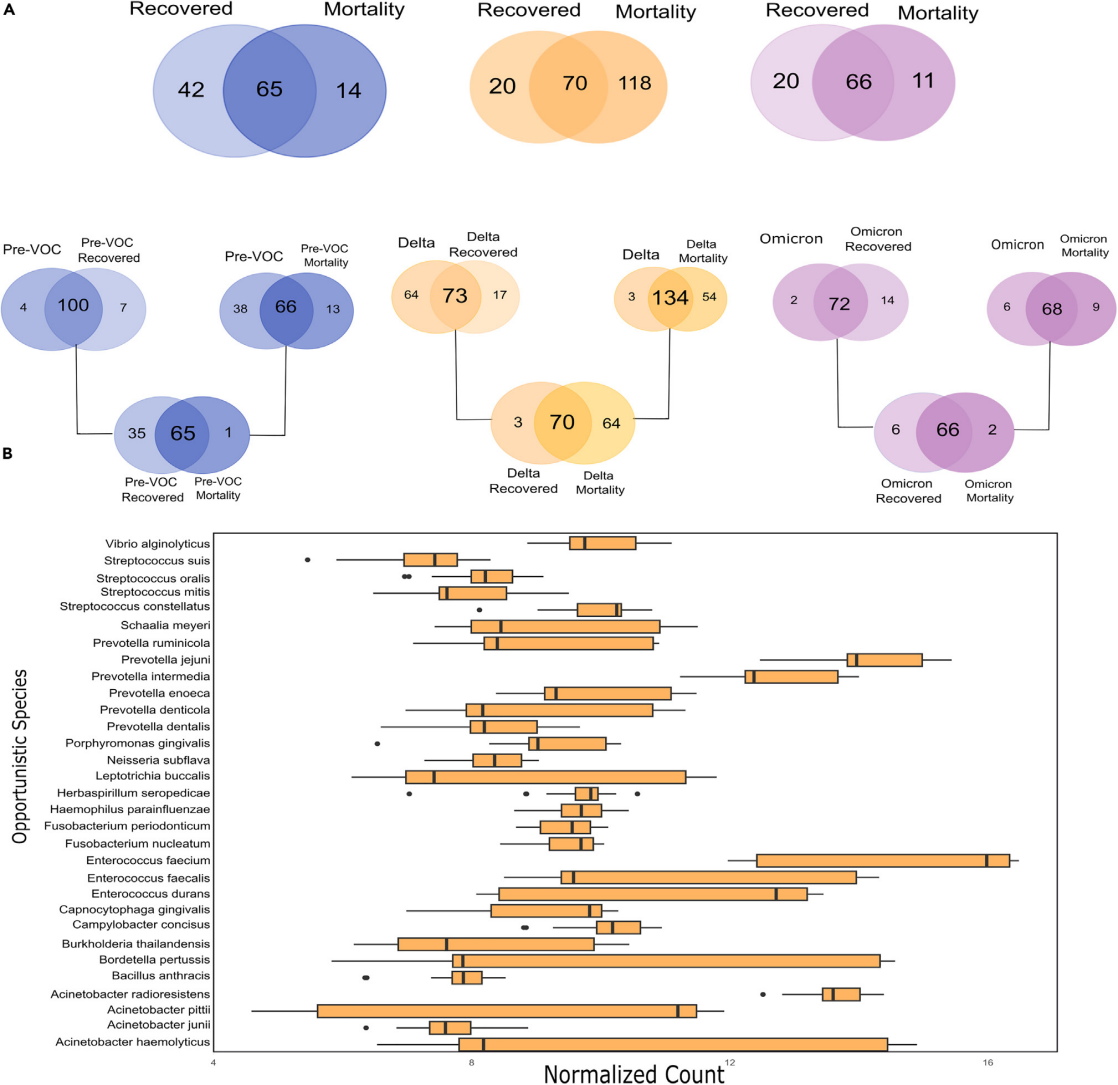
Microbial composition associated with clinical outcome, recovered or mortality for Pre-VOC, Delta, and Omicron (A) Venn diagram illustrating the numbers of shared and unique microbes, first between the Pre-VOCs/VOCs groups and their respective clinical outcome group, and then between the recovered and mortality group. (B) Plot representing the major bacterial species enriched in the Delta mortality patients.

Similar to the pattern observed in previous results between different variants, we noted an increase in diversity as we transitioned from Pre-VOC to VOCs for both recovery and mortality; however, more dispersion was detected in the Pre-VOC. We focused on gaining insight at the species level to determine whether the TAMs profiles obtained for each variant subgroup from previous results, corroborate in determining the differential clinical outcomes associated with the SARS-CoV-2 variants. For this, first, we compared total Pre-VOC species with those obtained specifically for Pre-VOC recovered and mortality. An identical strategy was employed for Delta and Omicron subgroups as well, Figure 5A. The common species obtained across all comparison groups were then checked for their presence, either uniquely or commonly, across recovered and mortality of different variants (Figure 5A). Interestingly, we obtained 35 vs. 1 unique species in recovered and mortality of Pre-VOC, 3 vs. 64 for Delta and 6 vs. 2 unique species in Omicron recovered and mortality.

Abundance of unique species in the Pre-VOC recovered and Delta mortality, cajoled us to look closely at these subgroups for functional associations with differential clinical outcomes. We observed both commensal and opportunistic species in the Pre-VOC recovered, which primarily might have led to mild disease outcomes. Studies report that *L. lactis, S. dysgalactiae, Streptococcus thermophilus, Streptococcus epidermis,* and *Moraxella osloensis* benefit the host by producing probiotics and out-competing virulent pathogens, which were discovered to be amongst the unique species of Pre-VOC recovered. Opportunistic species harbored were *S. oralis, S. mitis, S. pneumoniae, P. fusca, P. scopos, S. meyeri* along with *L. buccalis,* majorly belonged to the genus *Streptococcus* and *Prevotella*, both of which are reportedly elevated in COVID-19 patients. Importantly, the Delta mortality subgroup was notably more diverse and had preponderance of opportunistic species, Figure 5B. Essentially, the major genera of the Delta subgroup *Acinetobacter, Enterococcus, Prevotella,* and *Streptococcus* are associated with Delta mortality rather than the Delta recovered. Furthermore, other possible pathogenic species were identified such as *B. anthracis, B. pertussis, H. seropedicae, V. alginolyticus, H. parainfluenzae,* indicating a plausible role of TAMs in higher incidence of severity and mortality in the Delta infected patients. In Omicron, we did not find any significant difference for the unique species. Cumulatively, it seems that dysbiosis between commensal and opportunistic microbes modulates disease progression from mild to severe, as seen in Delta.

## Discussion

Next-generation sequencing enabled us to characterize the evolution of SARS CoV-2, which led to the identification of several variants of the virus leading to global health crisis and humongous challenges. Emergence and circulation of various SARS CoV-2 variants across different time points during the pandemic have been a cause of concern throughout, due to its temperament to alter degree of severity and clinical outcomes extravagantly. This is continuing even today with Recombinants being the latest challenge leading to spurts of infections.

Numerous studies have discussed several contributing factors to heterogeneity in COVID-19 disease symptoms, but changes in TAMs in the nasopharynx vis-a-vis SARS-CoV-2 infection, especially variants of concern, remain inadequately understood. Our prior studies have lit up the dynamics of commensal and opportunistic TAMs at various phases of disease severity and the quicker turnaround rate of patients who had received vaccinations.^13^^,^^17^ To the best of our knowledge, this is the first study to characterize the TAMs composition and host-microbe dynamics across different variants of SARS-CoV-2, namely Pre-VOC, Delta, and Omicron, as well as the clinical outcomes of each group, recovery, and mortality.

As previously highlighted, the disparity in richness and/or diversity in different microbial community landscapes is linked to changes in the host response that can modulate disease progression.^18^ On similar lines, we observed a significant increment in alpha diversity during the transition from Pre-VOC to the emergence of the VOCs. Of note, we found interpersonal variation in Pre-VOC with a low overall species richness and uneven distribution in taxon-level compositions, along with variation at the phylum and genus level. This could be attributed to the introduction of widespread use of face masks, increased attention to social distancing, and lockdown effects (altered food patterns) which were highly prevalent during the initial phase of the COVID-19 pandemic i.e., during Pre-VOC period, and could have influenced the composition of the nasopharyngeal microbiota.

We elucidated sequentially the differences in TAMs’ abundance at phylum, genus, and species level for different SARS-CoV-2 variants, Pre-VOC, and VOCs including Delta and Omicron. A higher relative abundance of *Proteobacteria* in VOCs compared to the Pre-VOC, especially with *Pseudomonas* genus domination highlights a similar trend in VOCs with other severe lung diseases like asthma and COPD^19^ specifically during Delta, which overruled the Omicron for both *Proteobacteria* and *Pseudomonas* genus. Moreover, the common phyla of healthy humans, *Firmicutes* predominated the Pre-VOCs. Few genera within *Firmicutes, Lactobacillus, Bacillus*, and *Clostridium* species were equally abundant between Pre-VOC and VOCs, although, Omicron overruled Delta within VOCs in the case of *Firmicutes*, more so, when the species of *Bacillus*, as well as *Lactobacillus*, have been documented in protecting against pathogenic overgrowth by producing bacteriocins and other inhibitory substances.^20^ This notable trend in Pre-VOC, Delta, and Omicron at the phyla and genera levels reflects the importance of the differential presence of taxa among SARS CoV-2 variants and their association with mild severity in Pre-VOC and Omicron albeit poor outcomes in Delta.

It is documented that a dysbiotic microbiome majorly harbors pathobionts that reflect an upheaval in metabolic pathways that increase inflammation and a reduction in pathways known to have anti-inflammatory properties.^21^ The preliminary elucidation of phyla and genera distribution paved the way for further investigation of core and variable microbiome at the species level, together with analysis of metabolic pathways associated with each species. *Streptococcus pneumoniae, Haemophilus influenzae, S. aureus*, and *K. pneumoniae* were found to be part of nasal core microbiota, verifying the association of SARS-CoV-2 infection with an upheaval of common respiratory pathogens in the disbiotic microbiota. Studies have reported that co-infections and superinfections might occur leading to poor outcomes and increased mortality in SARS-CoV-2 infected patients, with *K. pneumoniae, Streptococcus pneumoniae*, and *S. aureus* as the most frequently identified bacteria.^22^^,^^23^ Moreover, *H. influenzae* in the nasopharynx fostered *rhinovirus* virulence by upregulating TLR-3 expression on pulmonary epithelial cells.^24^ Similarly, *K. pneumoniae* is widely considered to be the primary pathogenic bacterium in nosocomial lower respiratory tract infections that result in antibiotic resistance, which can inevitably lead to treatment failures and higher rates of mortality.^25^ Out of all respiratory pathogens mentioned above, *K. pneumoniae* features exclusively in Delta with a highly active metabolic role demonstrated by the major pathways (FASYN-ELONG-PWY, FERMENTATION-PWY, PWY-7664, SER-GLYSYN-PWY, PWY-6282), known to augment proinflammatory responses in the host, that have been similarly documented in cystic fibrosis patients.^26^ This also precedes the finding that although these pathobionts were observed to be commonly present in all three variant subgroups, yet only *K. pneumoniae* was functionally relevant in Delta with high metabolic activity, which substantiates the differential activity of microbes during Delta infection outcome. Thus, an assessment of the core microbiota and associated metabolic pathways confirms that, due to the differential activity of pathobionts, the Delta patients experienced a higher rate of inflammation as compared to the Pre-VOC and Omicron.

Furthermore, integrated analysis between microbiota and host transcriptome provided us with unprecedented insights into the relationships between nasopharyngeal TAMs and genes differentially expressed in the nasopharynx during SARS-CoV-2 infection. In Pre-VOC, the metabolically active microbes, *P. jejunii*, and *H. parainfluenzae* had the highest number of associations with the expressed host genes involved in the NFKB and TNF signaling pathways. Another study has reported *P. jejunii* and *H. parainfluenzae* belonged to the group of biomarker species identified for COVID-19 during the initial phase of the pandemic.^27^ The negative correlation of these TAMs with innate immune pathways highlights its effect in moderation of cytokine response, hence suggestive of a moderate clinical presentation during the Pre-VOC phase. Similarly, *A. baumannii, P. stutzeri,* and *P. aeruginosa* were biomarker TAMs for Delta maximally correlating with host-expressed genes, where they seem to influence positively to aggravate the severity of the COVID-19 progression. Several studies had already reported *A. baumannii, P. stutzeri,* and *P. aeruginosa,* as major causes of secondary infection in hospital admitted COVID-19 patients.^28^^,^^29^^,^^30^^,^^31^ Moreover, the significant disruption of TAMs in Delta was persistent across clinical outcomes of recovery and mortality too. Delta mortality patients had a substantial presence of opportunistic bacterial species belonging to *Acinetobacter, Enterococcus, Prevotella,* and *Streptococcus* genera, which was lacking in the Pre-VOC and Omicron. Several species from these genera are usually resistant to multiple antibiotics and can cause severe and often fatal due to bloodstream infections and pneumonia.^25^^,^^32^ For e.g., Acinetobacter species, particularly *A. baumannii*, are known to cause nosocomial or hospital-acquired infections, such as meningitis, respiratory tract infections (RTI), urinary tract infections (UTI), endocarditis, and bacteremia.^33^ Furthermore, *A. baumannii* has been identified as a secondary infection in COVID-19 patients and is associated with drug resistance, potentially contributing to the progression of COVID-19 disease.^29^

Additionally, *Prevotella* has been enriched in other respiratory infections, including pulmonary empyema, lung abscess, inhalation pneumonia, chronic otitis, and sinusitis.^34^^,^^35^^,^^36^ *Prevotella* has also been identified as one of the most prevalent bacterial populations in the oropharynx of HIV-positive patients. During COVID-19, the prevalence of *Prevotella* has been found to be higher in infected patients. There is evidence to suggest that this microbial population may potentially play a role in promoting viral replication and contributing to the clinical severity of COVID-19 cases, as indicated in the following studies.^37^ Similarly, the *Enterococcus* genus has also been found to be predominant in COVID-19 patients, signifying its potential involvement in the disease process.^38^ These findings emphasize that microbial composition dysbiosis was more prevalent in the Delta infected patients and its mortality outcomes.

Contrarily, Omicron VOC, did not elaborate on the significant role of TAMs in the COVID-19 clinical response which coincides with the highly mild presentation of the Omicron variant. This could be attributed to several factors like, during a later wave, vaccines against the SARS-CoV-2 virus were successful in rapidly inoculating populations worldwide, as well as, a large population was already naturally infected with the SARS-CoV-2 till the time of the Omicron wave; favoring the immune system to be better equipped to deal with the challenges posed by the virus and secondary infections caused by opportunistic pathobionts of the microbiota.

In this context a thought-provoking question arise as to whether these bacteria can be targeted for therapeutic interventions? Several studies in literature have reported different ways for intervention. Firstly, targeting/manipulating the composition of dysbiotic microbiota through supplementation with bacterial substrates (prebiotics), live bacteria (probiotics) or inanimate bacterial preparations (postbiotics) has been proposed as a novel prevention and intervention strategy for infectious and chronic lung disease.^39^ Moreover, Oral biotherapeutic products (LBP) composed of mixtures of protective commensal bacterial strains have demonstrated impressive preclinical results in IBD (Inflammatory bowel disease).^40^ Secondly, selective depletion of opportunistic pathogens has also been considered as an alternative. For instance, utilising engineered *E. coli* with “sense-and-kill” system dependent on quorum sensing, it is possible to target *P. aeruginosa*, a known human pathogen.^41^ Combination therapy of colistin with sulbactam has also been recommended as the optimal treatment for severe infections caused by *A. baumannii*.^42^ In addition, selective depletion of opportunistic pathogens has also been reported through antibacterial monoclonal antibodies or bacteriophages, which has shown promising results in preventing bacterial pneumonia in at-risk patients.^39^ Since, we observed a differential presence of variable bacterial species in respective groups of Pre-VOC, Delta, and Omicron, targeting the bacteria specifically may be helpful in combating the course of disease and modulating the disease outcome.

### Conclusion

Our study revealed functional changes in the composition of TAMs in the COVID-19 patients infected during different pandemic waves in India - Pre-VOC, Delta, and Omicron, along with the clinical outcomes, recovery, and mortality. It was intriguing to discover that the respiratory pathogens, *Streptococcus pneumoniae, Haemophilus influenzae, S. aureus*, and *K. pneumoniae*, were part of a set of TAMs (core species) which demonstrated a common presence across all three variant subgroups, indicative of its presence due to SARS-CoV-2 infection. Notably, Delta deviated substantially from the Pre-VOC and Omicron, during variable species analysis, carrying a higher percentage of opportunistic species derived from the genus *Pseudomonas* and *Acinetobacter,* which plausibly attributes to its severe clinical manifestation. As we dug deeper into the mortality subgroups, we came across similar findings in Delta mortality, with several genera (*Acinetobacter, Enterococcus, Prevotella*) predominant in opportunistic pathogens, except for the presence of Pseudomonas, which may contribute to disease severity across the Delta variant rather than playing a role in modulating the clinical outcome. Our systematic, integrative study provides a window into the microbiome, host transcriptome, and their associations across the SARS-CoV-2 variants. Our research emphasized that there is a differential presence of variable bacterial species in respective groups of Pre-VOC, Delta, and Omicron, which correlated uniquely with expressed host genes and immune pathways, which helped to define the severity in Pre-VOC, Delta, and Omicron. Taken together, our findings suggest that the nasopharyngeal TAMs could be a factor potentially associated with the clinical manifestation of different variants, as highlighted by the observed predominance of opportunistic TAMs and enriched functionally relevant metabolic pathways in Delta. Additionally, the subsequent distribution of TAMs in the Delta mortality subgroup strengthens the rationale behind the increased severity and poor outcomes during the Delta wave.

### Limitations of the study

Due to the prevailing clinicians’ workload and clinical management challenges of high infection rates during the COVID-19 pandemic, our study was constrained toward collecting a comprehensive set of clinical parameters, particularly lacking comorbidities data in the Delta variant for our cohort. Incidentally, Delta was the most severe of the SARS-CoV-2 waves in India. We acknowledge that the clinical samples were primarily sourced from a single hospital and a specific region, which provides the advantage of lower variability between the samples but restricts the generalizability of our findings. To gain a more profound and inclusive understanding, future investigations should encompass multiple hospitals from diverse regions. We aim to deepen our knowledge with future investigations beyond COVID-19 and explore other RNA virus infectious diseases, such as dengue (DENV-1, DENV-2, DENV-3, and DENV-4), as well as influenza (types A, B, C, and D). This will provide a more comprehensive perspective on infectious diseases and their impact on diverse populations.

## STAR★Methods

### Key resources table

**Table:** 

REAGENT or RESOURCE	SOURCE	IDENTIFIER
Viral Transport Medium (VTM)	HiViral Transport Kit, HiMedia,	Cat. No: MS2760A-50NO
Viral RNA extraction	QIAmp viral mini kit, Qiagen	Cat. No. 52906
TRUPCR SARS-CoV-2 kit	3B BlackBio Biotech India Ltd	Cat. No. 3B304
TruSeq® Stranded Total RNA Library Prep Gold	Illumina	Cat. No. 20020599
AMPure XP	Beckman Coulter	Cat. No. A63881
Agencourt RNAClean XP Kit	Beckman Coulter	Cat. No. A63987
Qubit dsDNA HS Assay kit	Symbio (Thermo Fisher Scientific)	Cat. No. Q32854
Agilent 2100 Bioanalyzer	Agilent	Cat. No. 5067-4626

**Deposited data**		

RNA-seq data	This study, NCBI Sequence Read Archive (SRA) database	BioProject ID: PRJNA676016, PRJNA678831 (Pre-VOC), PRJNA868733, PRJNA952815 (VOCs)

**Software and algorithms**		

bcl2fastq	NA	GitHub - brwnj/bcl2fastq: NextSeq specific bcl2fastq2 wrapper.
FastQC	FastQC: a quality control tool for high throughput sequence data – ScienceOpen et al.^43^	Babraham Bioinformatics - FastQC A Quality Control tool for High Throughput Sequence Data
Trimmomatic v0.39	Bolger et al.^44^	USADELLAB.org - Trimmomatic: A flexible read trimming tool for Illumina NGS data
HISAT2	Kim et al.^45^	GitHub - DaehwanKimLab/hisat2: Graph-based alignment (Hierarchical Graph FM index)
Samtools	Danecek et al.^46^	https://github.com/samtools/
Kraken2	Wood et al.^47^	https://github.com/DerrickWood/kraken2/wiki
Bracken2	Lu et al.^48^	https://github.com/jenniferlu717/Bracken
metagenomeSeq	NA	https://github.com/HCBravoLab/metagenomeSeq
Vegan & Phyloseq	McMurdie, Dixon et al.^49^^,^^50^	https://joey711.github.io/phyloseq/tutorials-index.html

### Resource availability

#### Lead contact

Further information and requests for resources and reagents should be directed to and will be fulfilled by the lead contact, Rajesh Pandey (rajeshp@igib.in).

#### Materials availability

This study did not generate new unique reagents and material.

### Experimental model and study participant details

#### Study design

The study included 214 hospitalized patients with confirmed SARS-CoV-2 infection from three different time periods: April to July 2020, March to April 2021, and January to March 2022. The patients were selected based on a positive real-time reverse transcription-polymerase chain reaction (RT-PCR) test and whole genome sequencing to represent three SARS-CoV-2 variant groups, namely Pre-VOC, Delta, and Omicron. In this study, median age of the patients was 58 years in the Pre-VOC, 64 years in Delta and the 40.5 years in Omicron and gender (M/F ratio) among the three subgroups was comparable. All the patient samples were given anonymous barcodes at the CSIR-IGIB. Comprehensive clinical presentation and demographic data were also recorded electronically. Institutional ethical clearance for the study was obtained from both CSIR-IGIB and the Max hospital. The studies involving human participants were reviewed and approved by CSIR-IGIB’s Human Ethics Committee Clearance (Ref No: CSIR-IGIB/IHEC/2020-21/01). The patients/participants provided their written informed consent to participate in this study.

#### Collection and classification of clinical samples

The paramedical hospital staff collected the nasopharyngeal swab samples in Viral Transport Medium (VTM) (HiViral Transport Kit, HiMedia). Patients were categorized into three groups based on the infected SARS-CoV-2 variants (Pre-VOC, Delta, and Omicron) and further classified into the clinical outcome: recovered and mortality subgroups for each of the variant groups.

### Method details

#### RNA extraction, library preparation, and sequencing

For each clinical sample, viral RNA was extracted using QIAmp viral mini kit, Qiagen,and SARS-CoV-2 detection and quantification was performed using TRUPCR SARS-CoV-2 kit (3B BlackBio Biotech India Ltd.) with a cycle threshold of 35.

The library preparation was done using 250 ng of total RNA for each sample. The protocol for Holo-transcriptome using Illumina TruSeq Stranded Total RNA Library Prep Gold has been previously published from our lab. In brief, cytoplasmic and mitochondrial rRNA were removed using biotinylated target-specific oligos with Ribo-Zero rRNA removal beads. The purified RNA was fragmented using divalent cations under elevated temperature. The cDNA synthesis includes first strand cDNA prepared from the cleaved RNA fragments using reverse transcriptase and random primers, followed by second strand synthesis using DNA polymerase 1 and RNase H. The blunt 3′ end of the double-stranded cDNA was adenylated, followed by the addition of indexes and final amplification to enrich the library. The final library was purified using AMPure XP (Beckman Coulter). Libraries were quantified using the Qubit dsDNA HS Assay kit (Thermo Fisher Scientific). The cDNA libraries which passed the quality control analysis by the Agilent 2100 Bioanalyzer were used for sequencing on the NextSeq 2000 at 2 × 151 read length at a final loading concentration of 650 p.m.

#### Identification and segregation of human RNA reads from *meta*-transcriptomic data

Basecalls generated by the Illumina sequencing were converted into standard compressed FASTQ file format by bcl2fastq for downstream analysis. For each sample, FastQC^51^ was used to check the quality of the raw reads, and adapter and low-quality sequences were removed using Trimmomatic v0.39.^44^ This raw data contained reads from humans as well as microbes. Human-derived reads were identified by aligning the sequences using the HISAT2 v2.2.1^45^ algorithm onto the human reference genome GRCh38. The aligned human RNA reads were then removed to generate qualified non-human RNA-seq data using Samtools 1.17.^46^

#### Taxonomic classification of transcriptionally active microbes

Non-human reads were extracted for the *meta*-transcriptome analysis. The human unaligned sequences used for the taxonomic classification of microbial communities were inferred using Kraken2^47^ and Bracken2^48^ as per our previous study by Devi et al.^13^ Briefly, Kraken2, a taxonomic classifier was used to map with taxa using k-mers from the genomic database and assign microbial communities/taxonomy to the reads. The database was downloaded from the Kraken2 Website, which contains bacteria, archaea, and viral reference sequences. Although Kraken2 provides faster and more accurate classification, it cannot assign each read to the species. To overcome this problem, we analyzed Kraken2 output with Bracken2 (Bayesian Re-estimation of Abundance after classification with KrakEN), which uses the taxonomy assigned by Kraken2 to estimate the number of reads per sample that come from different species, using the Kraken report output file.^13^^,^^14^^,^^15^^,^^16^^,^^17^ The Kraken2 database was utilized to construct a Bracken-compatible database using the Bracken build function. An in-house Python program was written to combine the output from the bracken report file and add lineage information and convert it into a biom file using Kraken-biome to import into R for further analysis. To normalize the sampling depth, CSS (Cumulative Sum Scaling: median-like quantile normalization) method was used from R-package metagenomeSeq.^52^ Alpha and beta diversity analyses were executed in R (4.2.0) using vegan (v2.6-2)^49^ and Phyloseq (v1.40.0).^50^ Briefly, the estimate richness function was used to obtain alpha diversity (Shannon’s, and Chao-1) from the phyloseq package while phyloseq:distance from the vegan package was used to generate Bray-Curtis dissimilarity matrix and principal coordinate (PCoA) values. Afterward, the analysis data was exported from R as a CSV file and plotting and visualization was done in Python (3.6) using the library Seaborn (0.12.2) and Matplotlib (3.1) built on NumPy (1.11.0).

#### Functional enrichment analysis for Pre-VOC, Delta, and Omicron

HUMAnN3 (The Human Microbiome Project Unified Metabolic Analysis Network 3)^53^ analysis pipeline was used to illustrate the microbial population of the metabolic pathways based on the MetaCyc database. Humann_renorm_table was used to obtain normalized pathway abundance from RPK (Reads per kilobase) to CPM (counts per million) and joined tables using humann_join_tables. Pathways for common and unique bacterial species (analyzed by Kraken analysis) were extracted for separate groups of pre-VOC, Delta, and Omicron. Subsequently, microbial abundance along with the functional pathways were filtered based on the reads present in more than 20% of the samples per group to reduce the biases caused by the presence of zero. The top 20 shortlisted pathways were plotted in RCircos.^54^ Unique species pathways were extracted along with the bacteria and filtered out with the same parameters done for common bacterial species present in more than 20% of the samples.

#### Host transcriptome analysis

Raw reads were processed for quality check using FastQC v0.11.9, followed by trimming of adapter sequences using Trimmomatic v0.40. Reads were mapped to the human reference transcriptome (GENCODE) using Salmon quasi mapping tool to quantify transcript read abundance. We then used feature count to quantify the gene expression value. Differential gene expression analysis was performed using DESeq2^55^ between two variant groups, Pre-VOC vs. VOCs and Omicron vs. Delta.

#### Host-microbe interactions

To identify the correlation between bacterial species and the expressed host genes, unique species along with their metabolic pathways were extracted and filtered with abundance presence in more than 20% of the samples. To identify significant differentially expressed genes (DEGs), Wald’s test with a cut-off of p-adjusted value of ≤0.05, and Log2 fold change of ≥ ± 2 was applied. Gene Set Enrichment analysis (GSEA) was carried out with significant DEGs (FDR ≤0.05). The microbial species were utilized for correlation analysis with differentially expressed host genes using the corrr (0.4.4) package in R using Spearman’s correlation analysis. The cut-off for a correlation coefficient as ≥±0.8 and p value ≤0.05 was taken to build and visualize the interaction network in Cytoscape (version 3.9.1).^56^ All the analyses and data visualization were performed in R V4.2.0 with the following packages: phyloseq, vegan, tidyverse, dplyr, Adonis2, ggplot2, ggpubr, circlize, cytoscape and Python (3.6) using the library Seaborn (0.12.2) and Matplotlib (3.1) built on NumPy (1.11.0).

### Quantification and statistical analysis

To compare clinical significance along groups’ continuous numerical variables were compared as Mann–Whitney test. Sampling depth normalization of microbial count has been done using CSS (Cumulative Sum Scaling: median-like quantile normalization) method from R-package metagenomeSeq. Kruskal Wallis test used to calculate diversity richness significance of alpha diversity (Shannon and Chao-1; ‘∗∗∗∗’ p *value* < 0.0001; Figures 2A-i and 2A–ii) while permutational multivariate ANOVA (PERMANOVA) used to acquire beta diversity significance (Figure 2A-iii). Correlation analysis and significance calculation for genera level differences were done using Spearman’s Correlation with ‘∗’, ‘∗∗’, ‘∗∗∗’ signifying p *value* < 0.05, <0.01, <0.001 respectively (Figure 2B). To identify significant differentially expressed gene (DEGs), Wald’s test was applied (Figure 4B). The microbial species were used for correlation analysis with differentially expressed host genes using Spearman’s correlation analysis (Figure 4B). All the statistical analyses and data visualization was performed in R V4.2.0 with the following packages: phyloseq, vegan, tidyverse, dplyr, Adonis2, ggplot2, ggpubr, circlize, cytoscape and Python (3.6) using the library Seaborn (0.12.2) and Matplotlib (3.1) built on NumPy (1.11.0).

## Data Availability

• RNA-seq data have been deposited at NCBI SRA, and are publicly available as of the date of publication. Accession numbers are listed in the key resources table. All the data reported in this paper will be shared by the lead contact upon request. • This paper does not report original code. • Any additional information required to reanalyze the data reported in this paper is available from the lead contact upon request.

## Supplemental information

Document S1. Figures S1–S3

Table S1. Distribution of microbial and human reads for all samples from the RNA-Seq dataset, related to Figure 1

Table S2. Alpha diversity indices showing bacterial species richness, and abundance across the patients in Pre-VOC, VOCs, Delta and Omicron cohort, related to Figure 2

Table S3. Taxonomic communities at the level of phylum identified through Bracken in Pre-VOC, VOCs, Delta and Omicron cohort, related to Figure 2

Table S4. A total of 71 genera were detected which contain variable number of species across all four groups, related to Figure 3B

Table S5. Metabolic pathways associated with variable microbes across the groups (Pre-VOC, Delta, Omicron), related to Figure 4B

Table S6. MetaCYC database pathway function analysis, related to Figure 4A

Table S7. DEGs list representing the transcriptome file between Pre-VOC and VOCs, related to Figure 4B And DEGs list representing the transcriptome file between Delta and Omicron.

Table S8. Host Pathways Enrichment through GSEA across Pre-VOC and VOCs, related to Figure 4B And Host Pathways Enrichment through GSEA across Delta and Omicron.

Table S9. Alpha diversity (Shannon H & Chao-1) indices for Pre-VOC, Delta and Omicron, related to Figure 2

Table S10. Taxonomic communities (Phylum and Genus) for Pre-VOC, Delta and Omicron categorized by clinical outcomes (Recovered and Mortality), related to Figure 5

## References

[1] Cucinotta D., Vanelli M. (2020). WHO Declares COVID-19 a Pandemic. Acta Biomed..

[2] Grimaldi A., Panariello F., Annunziata P., Giuliano T., Daniele M., Pierri B., Colantuono C., Salvi M., Bouché V., Manfredi A. (2022). Improved SARS-CoV-2 sequencing surveillance allows the identification of new variants and signatures in infected patients. Genome Med..

[3] Carabelli A.M., Peacock T.P., Thorne L.G., Harvey W.T., Hughes J., Peacock S.J., Barclay W.S., de Silva T.I., Towers G.J., Robertson D.L., COVID-19 Genomics UK Consortium (2023). SARS-CoV-2 variant biology: immune escape, transmission and fitness. Nat. Rev. Microbiol..

[4] Wrenn J.O., Pakala S.B., Vestal G., Shilts M.H., Brown H.M., Bowen S.M., Strickland B.A., Williams T., Mallal S.A., Jones I.D. (2022). COVID-19 severity from Omicron and Delta SARS-CoV-2 variants. Influenza Other Respi. Viruses.

[5] Ahmad A., Fawaz M.A.M., Aisha A. (2022). A comparative overview of SARS-CoV-2 and its variants of concern. Infezioni Med. Le.

[6] Butt A.A., Dargham S.R., Chemaitelly H., Al Khal A., Tang P., Hasan M.R., Coyle P.V., Thomas A.G., Borham A.M., Concepcion E.G. (2022). Severity of Illness in Persons Infected With the SARS-CoV-2 Delta Variant vs Beta Variant in Qatar. JAMA Intern. Med..

[7] Ao D., Lan T., He X., Liu J., Chen L., Baptista-Hon D.T., Zhang K., Wei X. (2022). SARS-CoV-2 Omicron variant: Immune escape and vaccine development. MedComm.

[8] Zsichla L., Müller V. (2023). Risk Factors of Severe COVID-19: A Review of Host, Viral and Environmental Factors. Viruses.

[9] Schultze J.L., Aschenbrenner A.C. (2021). COVID-19 and the human innate immune system. Cell.

[10] Bajaj V., Gadi N., Spihlman A.P., Wu S.C., Choi C.H., Moulton V.R. (2020). Aging, Immunity, and COVID-19: How Age Influences the Host Immune Response to Coronavirus Infections?. Front. Physiol..

[11] Gupta A., Bhanushali S., Sanap A., Shekatkar M., Kharat A., Raut C., Bhonde R., Shouche Y., Kheur S., Sharma A. (2022). Oral dysbiosis and its linkage with SARS-CoV-2 infection. Microbiol. Res..

[12] Pérez-Losada M., Graham R.J., Coquillette M., Jafarey A., Castro-Nallar E., Aira M., Hoptay C., Freishtat R.J., Mansbach J.M. (2018). Tracheal microbiota in patients with a tracheostomy before, during and after an acute respiratory infection. Pediatr. Infect. Dis. J..

[13] Devi P., Kumari P., Yadav A., Tarai B., Budhiraja S., Shamim U., Pandey R. (2023). Transcriptionally active nasopharyngeal commensals and opportunistic microbial dynamics define mild symptoms in the COVID 19 vaccination breakthroughs. PLoS Pathog..

[14] Rafiqul Islam S.M., Foysal M.J., Hoque M.N., Mehedi H.M.H., Rob M.A., Salauddin A., Tanzina A.Y., Biswas S., Noyon S.H., Siddiki A.M.A.M.Z. (2022). Dysbiosis of Oral and Gut Microbiomes in SARS-CoV-2 Infected Patients in Bangladesh: Elucidating the Role of Opportunistic Gut Microbes. Front. Med..

[15] Liu T.F.D., Philippou E., Kolokotroni O., Siakallis G., Rahima K., Constantinou C. (2022). Gut and airway microbiota and their role in COVID-19 infection and pathogenesis: a scoping review. Infection.

[16] Hoque M.N., Rahman M.S., Sarkar M.M.H., Habib M.A., Akter S., Banu T.A., Goswami B., Jahan I., Hossain M.A., Khan M.S., Islam T. (2023). Transcriptome analysis reveals increased abundance and diversity of opportunistic fungal pathogens in nasopharyngeal tract of COVID-19 patients. PLoS One.

[17] Devi P., Maurya R., Mehta P., Shamim U., Yadav A., Chattopadhyay P., Kanakan A., Khare K., Vasudevan J.S., Sahni S. (2022). Increased Abundance of Achromobacter xylosoxidans and Bacillus cereus in Upper Airway Transcriptionally Active Microbiome of COVID-19 Mortality Patients Indicates Role of Co-Infections in Disease Severity and Outcome. Microbiol. Spectr..

[18] Blanco-Fuertes M., Correa-Fiz F., Fraile L., Sibila M., Aragon V. (2021). Altered Nasal Microbiota Composition Associated with Development of Polyserositis by Mycoplasma hyorhinis. Pathogens.

[19] Rizzatti G., Lopetuso L.R., Gibiino G., Binda C., Gasbarrini A. (2017). Proteobacteria: A common factor in human diseases. BioMed Res. Int..

[20] Bogaert D., Keijser B., Huse S., Rossen J., Veenhoven R., van Gils E., Bruin J., Montijn R., Bonten M., Sanders E. (2011). Variability and diversity of nasopharyngeal microbiota in children: a metagenomic analysis. PLoS One.

[21] Haran J.P., Bradley E., Zeamer A.L., Cincotta L., Salive M.-C., Dutta P., Mutaawe S., Anya O., Meza-Segura M., Moormann A.M. (2021). Inflammation-type Dysbiosis of the Oral Microbiome Associates with the Duration of COVID-19 Symptoms and Long COVID. JCI Insight.

[22] Giugliano R., Sellitto A., Ferravante C., Rocco T., D’Agostino Y., Alexandrova E., Lamberti J., Palumbo D., Galdiero M., Vaccaro E. (2022). NGS analysis of nasopharyngeal microbiota in SARS-CoV-2 positive patients during the first year of the pandemic in the Campania Region of Italy. Microb. Pathog..

[23] Gauthier N.P.G., Locher K., MacDonald C., Chorlton S.D., Charles M., Manges A.R. (2022). Alterations in the nasopharyngeal microbiome associated with SARS-CoV-2 infection status and disease severity. PLoS One.

[24] Sajjan U.S., Jia Y., Newcomb D.C., Bentley J.K., Lukacs N.W., LiPuma J.J., Hershenson M.B. (2006). H. influenzae potentiates airway epithelial cell responses to rhinovirus by increasing ICAM-1 and TLR3 expression. Faseb. J..

[25] Mancuso G., Midiri A., Gerace E., Biondo C. (2021). Bacterial antibiotic resistance: the most critical pathogens. Pathogens.

[26] Felton E., Burrell A., Chaney H., Sami I., Koumbourlis A.C., Freishtat R.J., Crandall K.A., Hahn A. (2021). Inflammation in children with cystic fibrosis: contribution of bacterial production of long-chain fatty acids. Pediatr. Res..

[27] Iebba V., Zanotta N., Campisciano G., Zerbato V., Di Bella S., Cason C., Luzzati R., Confalonieri M., Palamara A.T., Comar M. (2021). Profiling of Oral Microbiota and Cytokines in COVID-19 Patients. Front. Microbiol..

[28] Ceparano M., Baccolini V., Migliara G., Isonne C., Renzi E., Tufi D., De Vito C., De Giusti M., Trancassini M., Alessandri F. (2022). Acinetobacter baumannii Isolates from COVID-19 Patients in a Hospital Intensive Care Unit: Molecular Typing and Risk Factors. Microorganisms.

[29] Rangel K., Chagas T.P.G., De-Simone S.G. (2021). Acinetobacter baumannii Infections in Times of COVID-19 Pandemic. Pathogens.

[30] Rayeesa Faheem S., Srinivas T., Sadhana Y. (2022). A Comparative Study of Acinetobacter Infections in COVID and Non-COVID Patients. J. Infect. Dis. Epidemiol..

[31] Rhoades N.S., Pinski A.N., Monsibais A.N., Jankeel A., Doratt B.M., Cinco I.R., Ibraim I., Messaoudi I. (2021). Acute SARS-CoV-2 infection is associated with an increased abundance of bacterial pathogens, including Pseudomonas aeruginosa in the nose. Cell Rep..

[32] Chiș A.A., Rus L.L., Morgovan C., Arseniu A.M., Frum A., Vonica-Țincu A.L., Gligor F.G., Mureșan M.L., Dobrea C.M. (2022). Microbial resistance to antibiotics and effective antibiotherapy. Biomedicines.

[33] Gedefie A., Demsis W., Ashagrie M., Kassa Y., Tesfaye M., Tilahun M., Bisetegn H., Sahle Z. (2021). Acinetobacter baumannii Biofilm Formation and Its Role in Disease Pathogenesis: A Review. Infect. Drug Resist..

[34] Ito K., Ito Y., Mizuta K., Ogawa H., Suzuki T., Miyata H., Kato N., Watanabe K., Ueno K. (1995). Bacteriology of chronic otitis media, chronic sinusitis, and paranasal mucopyocele in Japan. Clin. Infect. Dis..

[35] Zhu T., Yang W., Lu W. (2023). Risk factors associated with length of hospital stay and medical expenses in pulmonary abscess patients: retrospective study. PeerJ.

[36] Tamanai-Shacoori Z., Le Gall-David S., Moussouni F., Sweidan A., Polard E., Bousarghin L., Jolivet-Gougeon A. (2022). SARS-CoV-2 and Prevotella spp.: friend or foe? A systematic literature review. J. Med. Microbiol..

[37] Xiong D., Muema C., Zhang X., Pan X., Xiong J., Yang H., Yu J., Wei H. (2021). Enriched Opportunistic Pathogens Revealed by Metagenomic Sequencing Hint Potential Linkages between Pharyngeal Microbiota and COVID-19. Virol. Sin..

[38] Bhonchal Bhardwaj S., Kırmusaoğlu S., Bhonchal Bhardwaj S. (2020). Pathogenic Bacteria.

[39] Mindt B.C., DiGiandomenico A. (2022). Microbiome modulation as a novel strategy to treat and prevent respiratory infections. Antibiotics.

[40] Oka A., Sartor R.B. (2020). Microbial-Based and Microbial-Targeted Therapies for Inflammatory Bowel Diseases. Dig. Dis. Sci..

[41] Saeidi N., Wong C.K., Lo T.-M., Nguyen H.X., Ling H., Leong S.S.J., Poh C.L., Chang M.W. (2011). Engineering microbes to sense and eradicate Pseudomonas aeruginosa, a human pathogen. Mol. Syst. Biol..

[42] Kengkla K., Kongpakwattana K., Saokaew S., Apisarnthanarak A., Chaiyakunapruk N. (2018). Comparative efficacy and safety of treatment options for MDR and XDR Acinetobacter baumannii infections: a systematic review and network meta-analysis. J. Antimicrob. Chemother..

[43] FastQC: a quality control tool for high throughput sequence data – ScienceOpen. https://www.scienceopen.com/document?vid=de674375-ab83-4595-afa9-4c8aa9e4e736.

[44] Bolger A.M., Lohse M., Usadel B. (2014). Trimmomatic: a flexible trimmer for Illumina sequence data. Bioinformatics.

[45] Kim D., Paggi J.M., Park C., Bennett C., Salzberg S.L. (2019). Graph-based genome alignment and genotyping with HISAT2 and HISAT-genotype. Nat. Biotechnol..

[46] Danecek P., Bonfield J.K., Liddle J., Marshall J., Ohan V., Pollard M.O., Whitwham A., Keane T., McCarthy S.A., Davies R.M., Li H. (2021). Twelve years of SAMtools and BCFtools. GigaScience.

[47] Wood D.E., Lu J., Langmead B. (2019). Improved metagenomic analysis with Kraken 2. Genome Biol..

[48] Lu J., Breitwieser F.P., Thielen P., Salzberg S.L. (2017). Bracken: estimating species abundance in metagenomics data. PeerJ Comput. Sci..

[49] Dixon P. (2003). VEGAN, a package of R functions for community ecology. J. Veg. Sci..

[50] McMurdie P.J., Holmes S. (2013). phyloseq: an R package for reproducible interactive analysis and graphics of microbiome census data. PLoS One.

[51] Babraham Bioinformatics - FastQC A Quality Control tool for High Throughput Sequence Data. https://www.bioinformatics.babraham.ac.uk/projects/fastqc/.

[52] Paulson J.N., Stine O.C., Bravo H.C., Pop M. (2013). Differential abundance analysis for microbial marker-gene surveys. Nat. Methods.

[53] Beghini F., McIver L.J., Blanco-Míguez A., Dubois L., Asnicar F., Maharjan S., Mailyan A., Manghi P., Scholz M., Thomas A.M. (2021). Integrating taxonomic, functional, and strain-level profiling of diverse microbial communities with bioBakery 3. Elife.

[54] Zhang H., Meltzer P., Davis S. (2013). RCircos: an R package for Circos 2D track plots. BMC Bioinf..

[55] Love M.I., Huber W., Anders S. (2014). Moderated estimation of fold change and dispersion for RNA-seq data with DESeq2. Genome Biol..

[56] Shannon P., Markiel A., Ozier O., Baliga N.S., Wang J.T., Ramage D., Amin N., Schwikowski B., Ideker T. (2003). Cytoscape: a software environment for integrated models of biomolecular interaction networks. Genome Res..

