# Supplementary material for: Longitudinal study across SARS-CoV-2 variants identifies transcriptionally active microbes (TAMs) associated with Delta severity
Source: iScience. 2023 Aug 29;26(10):107779. doi: 10.1016/j.isci.2023.107779 (PMC10493601; doi:10.1016/j.isci.2023.107779)
Supplement: Document S1. Figures S1–S3 [file mmc1.pdf]

iScience, Volume *26*

Supplemental information

Longitudinal study across SARS-CoV-2 variants identifies transcriptionally active microbes (TAMs) associated with Delta severity

Priti Devi, Pallawi Kumari, Aanchal Yadav, Bansidhar Tarai, Sandeep Budhiraja, Uzma Shamim, and Rajesh Pandey

Supplementary Figure S1

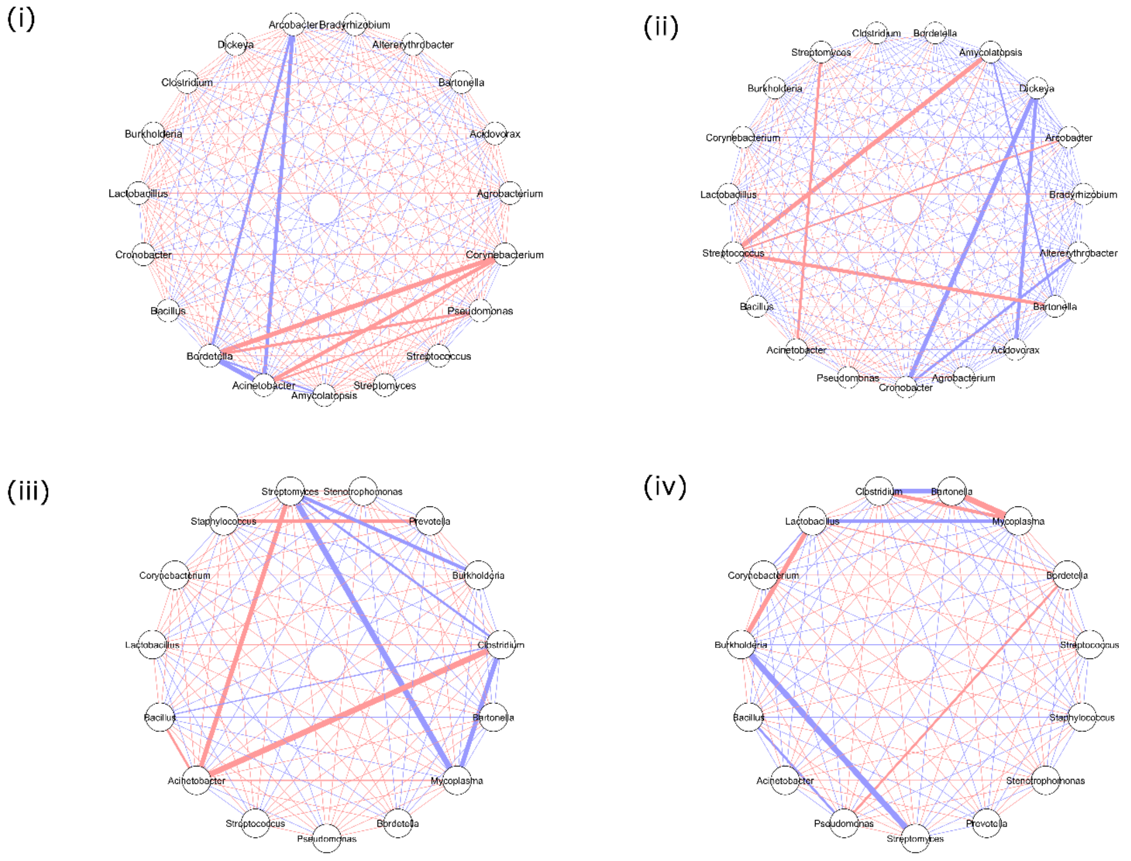

Figure S1: Visualization of co-occurrence network based on spearman correlation analysis between the different genera across different groups. Related to Figure 2. Specific interactions between the different genera of i) Pre-VOC, ii) VOCs, iii) Delta, iv) Omicron wherein the edges represent the strength of correlation and their colours shows positive and negative as blue and red, respectively.

Supplementary Figure S2

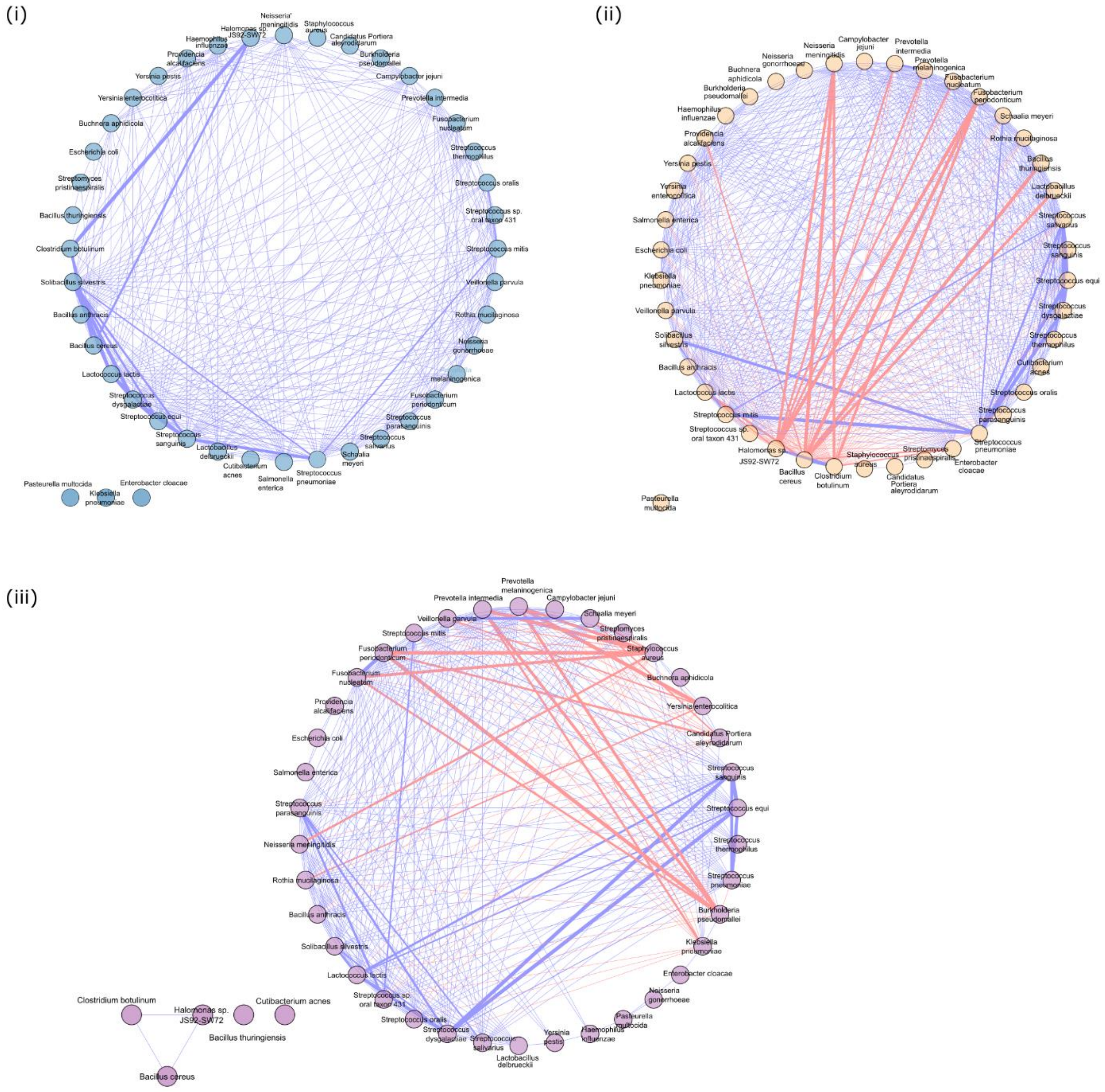

Figure S2: Visualization of co-occurrence network based on spearman correlation analysis between the core bacterial species across different groups shows significant ($p < 0.05$, $r > \pm 0.6$) specific interaction. Related to Figure 3A. i) Pre-VOC, ii) Delta, iii) Omicron wherein the edges represent the strength of correlation and their colors shows positive and negative as blue and red, respectively.

Supplementary Figure S3

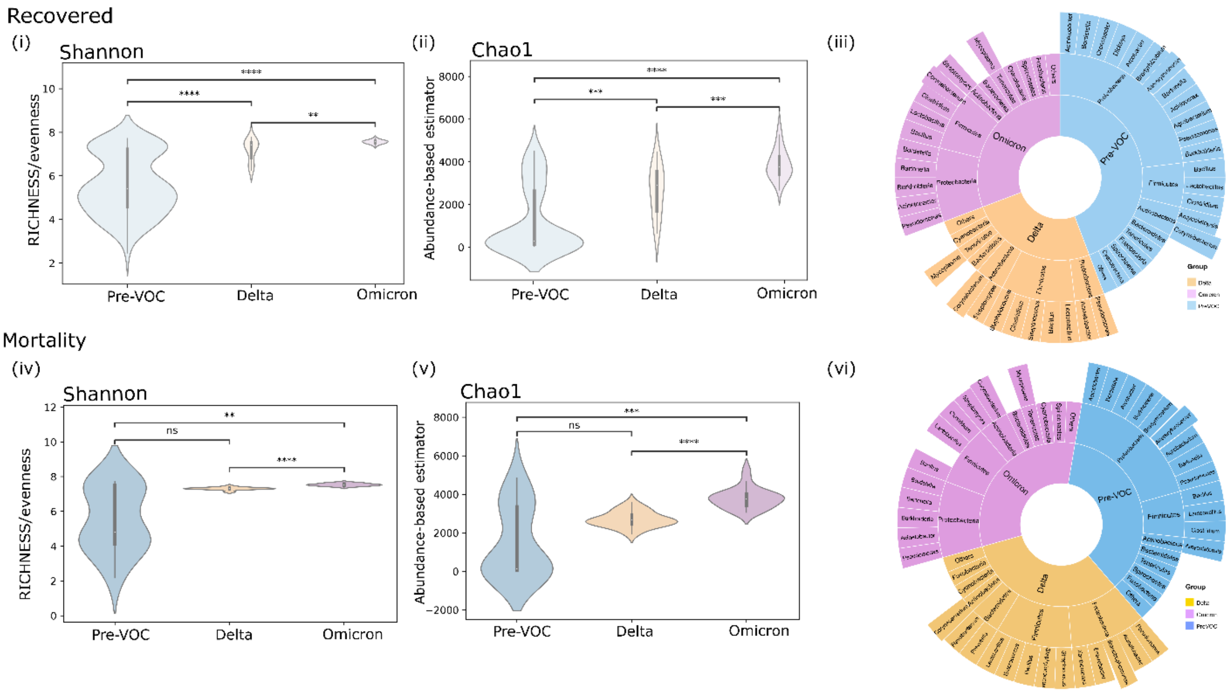

Figure S3: Figure illustrating the alpha diversity and abundance profile of phyla and genera for recovered and mortality sub-groups of Pre-VOC, Delta and Omicron. Related to Figure 5: i) Shannon (Richness), (ii) Chao-1 (Abundance-based estimator) with *p-values* calculated by the Kruskal Wallis test, iii) Visualization of percent relative abundance proportion of phyla and genera identified in recovered subgroup: iv) Shannon (Richness), (v) Chao-1 (Abundance-based estimator) with *p-values* calculated by the Kruskal Wallis test, vi) Visualization of percent relative abundance proportion of phyla and genera identified in mortality subgroup of Pre-VOC, Delta and Omicron.
